# The Effect of an External Magnetic Field on the Electrocatalytic Activity of Heat-Treated Cyanometallate Complexes towards the Oxygen Reduction Reaction in an Alkaline Medium

**DOI:** 10.3390/ma15041418

**Published:** 2022-02-14

**Authors:** Barbara Zakrzewska, Lidia Adamczyk, Marek Marcinek, Krzysztof Miecznikowski

**Affiliations:** 1 Faculty of Chemistry, University of Warsaw, Pasteura 1, 02-093 Warsaw, Poland; bzakrzewska@chem.uw.edu.pl; 2 Faculty of Production Engineering and Materials Technology, Czestochowa University of Technology, Al. Armii Krajowej 19, 42-201 Czestochowa, Poland; lidia.adamczyk@pcz.pl; 3 Faculty of Chemistry, Warsaw University of Technology, Noakowskiego 3, 00-664 Warsaw, Poland; markmar@ch.pw.edu.pl

**Keywords:** oxygen reduction reaction, functionalized reduced graphene oxide, Prussian blue, alkaline medium, electrocatalysis, magnetic field

## Abstract

This work focuses on the development of an electrocatalytic material by annealing a composite of a transition metal coordination material, iron hexacyanoferrate (Prussian blue) immobilized on carboxylic-acid-functionalized reduced graphene oxide. Pyrolysis at 500 °C under a nitrogen atmosphere formed nanoporous core–shell structures with efficient activity, which mostly included iron carbide species capable of participating in the oxygen reduction reaction in alkaline media. The physicochemical properties of the iron-based catalyst were elucidated using transmission electron microscopy, X-ray diffraction, Mössbauer spectroscopy, and various electrochemical techniques, such as cyclic voltammetry and rotating ring–disk electrode (RRDE) voltammetry. To improve the electroreduction of oxygen over the studied catalytic material, an external magnetic field was utilized, which positively shifted the potential by ca. 20 mV. The formation of undesirable intermediate peroxide species was decreased compared with the ORR measurements without an external magnetic field.

## 1. Introduction

The most challenging tasks facing humans are energy shortages and environmental pollution [1,2]. Hence, fuel cells have been applied to create efficient and ecofriendly technologies for converting chemical energy into electricity. Polymer electrolyte membrane fuel cells (PEMFCs) are one example of such devices, which use hydrogen or small organic compounds as the fuel, and oxygen as the oxidant, to produce electricity with water or CO_2_ as the major products [3]. The electrochemical reduction of oxygen (ORR) that takes place at the cathode is the limiting step in most fuel cells and in metal–air batteries, which is directly due to the sluggish ORR kinetics [4,5,6,7]. Electrocatalytic oxygen reduction can be enhanced in two ways: either by using more active catalysts (e.g., new catalyst) or using external process intensifications using different type of energy. In the first strategy, there is a need to develop new electrocatalysts for more efficient ORR that exhibit high selectivity toward the four-electron reduction of O_2_ to H_2_O, rather than to the two-electron reaction which produces undesirable H_2_O_2_. Platinum and Pt-based catalysts seem to be the best electrocatalytic materials for ORR because of their high activity in both acidic and alkaline media [8,9]. Sadly, the scarce resources, high prices, and decreased activity of Pt in the presence of small organic compounds (crossover effect in applications towards alcohol fuel cells) make them difficult to use in commercial applications. As a consequence, it is still challenging to develop efficient non-precious metal or metal-free catalysts for ORR [10,11,12,13,14,15,16,17,18].

In the last few decades, several concepts and precursors have been utilized to develop many advanced non-precious metal catalysts that show enhanced activity towards the electrochemical reduction of oxygen in both alkaline and acidic media. These materials include metal–organic frameworks [19,20,21,22], conductive-polymer-based complexes (pyrolyzed and non-pyrolyzed) [23,24], non-pyrolyzed transition metal macrocycles [25,26], materials based on metal oxides/carbides/nitrides [27,28], and heat-treated and untreated metal–nitrogen–carbon catalysts [29,30,31]. Recently, a curious approach emerged regarding transition metal–nitrogen–carbon electrocatalytic materials derived from the thermal decomposition of hexacyanometallates, where a carbon–nitrogen-based matrix coordinates metal centers [16,32,33,34,35,36]. Metal hexacyanometallates belong to a class of inorganic polymeric compounds where cyanide groups (–C≡N–) link the same or different transition metals ions. One of the most well-known and well-studied metal hexacyanometallates is iron (III) hexacyanoferrate (Prussian blue) and its analogues. An advantage of employing metal hexacyanometallates as a precursor is their ability to form a series of compounds composed of a variety of transition metal ion combinations coordinated by nitrogen or carbon atoms. The nitrogen and carbon atoms originate from the cyano ligands of simple transition metal salts, as well as from combining species (two independent redox states) to improve the catalytic activity for ORR [16,32]. Consequently, metal hexacyanometallates are promising precursors for the development of N-M-C catalysts with high electrocatalytic activities.

The second strategy uses external energy to enhance the ORR efficiency, e.g., an external magnetic field with a different power generated by permanent magnets. From a broader perspective, the role of magnetism in heterogeneous catalysis is underestimated, even though the reactivity of metal surfaces is dependent on their magnetic state. To understand how magnetic fields change the conditions of these chemical systems, it is necessary to learn more about the characteristics of magnetic energy and the associated forces [37,38,39]. Generally, there are two main magnetic forces in electrochemical processes that play a role in inducing these magnetic phenomena. Of these forces, one is dependent on the magnetic properties of the electrolyte species (Lorentz force—magnetohydrodynamic effect), and the second is dependent on the motion of paramagnetic species in the system (Kelvin force—magnetic field–gradient force) [37,38]. Both forces are usually present because any redox process where a single electron is transferred involves paramagnetic ions or free radicals with an unpaired spin. Wakayama et al. carried out work on the utilization of magnets to enhance the performance of proton exchange membrane fuel cells (PEMFC) [40]. In their work, they noted that the limiting current densities of the ORR on pristine platinum in an acidic medium in the presence of a magnetic field of 0.56 T was ca. 28% higher due to the attractive Kelvin force over paramagnetic O_2_. Wang et al. studied the effect of an external magnetic field on the ORR over ferromagnetic catalysts containing iron oxides and showed that the ORR currents increased with the strength of the magnetic field [41]. Coey et al. reported the effect of localized magnetic fields on the electroreduction of oxygen using microspheres of Fe, Co, and Zn [42,43,44]. The maximum oxygen reduction current was obtained for magnetized Fe and Co instead of Zn due to the presence of a stronger Kelvin force near the ferromagnetic species.

In this study, we used heat-treated Prussian blue immobilized on carboxylic-acid-functionalized reduced graphene oxide as a model iron-based catalyst and investigated its activity for the oxygen reduction reaction in alkaline media in the presence and absence of an external magnetic field. The catalytic activity of the resulting material was compared to the performance of benchmark Pt nanoparticles.

## 2. Materials and Methods

All chemicals were commercial available, and they were applied without further purification. KOH, potassium nitrate, and 2-propanol were purchased from Sigma-Aldrich. The iron (III) nitrate (Fe(NO_3_)_3_), potassium hexacyanoferrate (III) hydrate (K_3_[Fe(CN)_6_]), 5 wt% Nafion solution, and the carboxylic-acid-functionalized reduced graphene oxide (rGO-COOH) were also purchased from Merck (Darmstadt, Germany); 20% Pt/Vulcan nanoparticles (reference catalyst) was purchased from Premetek (Cherry Hill, NJ, USA). The used gases (nitrogen and oxygen) (purity 99.999%) were purchased from Air Products (Warsaw Poland). All solutions was prepared using triply distilled and deionized water. Preparation of iron hexacyanoferrate (Prussian blue—FeHCNFe)-modified, carboxylic-acid-functionalized reduced graphene-oxide-supported materials (rGO-COOH) was achieved by the wet-chemical aging process. In the typical procedure of synthesis of FeHCNFe-modified, carboxylic-acid-functionalized reduced graphene oxide (rGO-COOH) material; a known amount (100 mg) of rGO-COOH-supported materials was mixed with 5 cm^3^ of 0.01 mol dm^−3^ Fe(NO_3_)_3_ aqueous solution and held for 30 min in an ultrasonics bath to obtain water suspension. Afterwards, 5 cm^−3^ of 0.01 mol dm^−3^ K_3_[Fe(CN)_6_] aqueous solution was introduced, and the slurry was left under magnetic stirring at least for 12 h. After that, the precipitate was separated by centrifuging and washed with water (this step was iterated a number of times). Finally, the outcome slurry was dried at about 50 °C. The metal loading of Fe in the proposed catalyst system was determined based on considering the amounts of carbon-supported materials and precursors utilized for synthesis as well as used X-ray fluorescence (XRF); in both cases, the total content of iron in the catalytic material was consistent and amounted approximately 20%. The resulting powders were annealed under inert atmosphere (N_2_) in a glass quartz tubes for 2 h at 500 °C (with a 5 °C min^−1^ ramp). The heat-treatment sample was marked and abbreviated as CFeN@rGO-COOH.

To produce a catalytic inks containing CFeN@rGO-COOH, a known amount of the resulting catalyst (50 mg) was dispersed in 1 cm^−3^ of 2-propanol and Nafion (20% by weight) as solvent and binder, respectively. The resulting ink was exposed to an ultrasonic bath for 30 min then left on magnetic stirring for 24 h to get a homogeneous suspension. The slurry created in this procedure was featured by required homogeneity, as well as displayed lower tendency to agglomerate. Subsequently, a catalytic layer was made onto the surface of glassy carbon disk electrode by dropping 6 μL of the appropriate catalyst ink to attain the loading on the level of 300 μg cm^−2^ after vaporizing solvent. Prior to being utilized in electroreduction reaction, the prepared catalytic films were conditioned through the employment of full oxidation/reduction cycles in the potential range from 0 to 1 V (at 50 mV s^−1^) in 0.1 mol dm^−3^ KOH solution under nitrogen atmosphere until steady-state current responses were achieved.

The electrochemical measurements were performed using CH Instruments (Austin, TX, USA) Model 760E workstation in a standard three-electrode system. A mercury/mercury oxide electrode (Hg/HgO), which potential was 98 mV more negative relative to the reversible hydrogen electrode (RHE), was applied as a reference electrode. Prior to perform each experiments the potential of reference electrode was calibrated relative to a reversible hydrogen electrode (RHE) in the same supporting electrolyte. Herein, all potentials were expressed versus the reversible hydrogen electrode (RHE). To avoid any contamination from platinum, the carbon rod was applied as counter electrode. The rotating ring–disk electrode (RRDE-3A ver. 2.0) from ALS Co., Ltd. (Tokyo, Japan) was attended as working electrode and contained a glassy carbon disk (geometric area 0.197 cm^−2^) and a Pt ring. In order to apply the rotating ring–disk electrode to determine the activity of catalyst towards ORR, it is necessitated the knowledge of a parameter called the collection efficiency (N), which can be figured out theoretically by the radius of disk and ring as well as the spacing between them or experimentally based on the ration of ring current to disk current recording for a redox reaction of the Fe(CN)_6_^3−^/Fe(CN)_6_^4−^ couple. The experimental collection efficiency (N) was equal to 0.43 and it was determined based on 6 independent measurements. The electrode substrates were prepared by polishing with 5 μm successively and 0.05 μm aqueous alumina solutions on a Buehler polishing cloth. The potential of the ring electrode was held at 1.2 V (vs. RHE) during the electroreduction of oxygen at the disk electrode. The applied potential is sufficient to oxidize any formed peroxide ions and can be detected at Pt ring electrode. The RRDE experiments were recorded at a scan rate 10 mV s^−1^ and the obtained current values were converted into appropriated current densities with refer to the geometric surface area of disk electrode. Prior to each electrochemical experiments the electrolyte solution was deaerated (applying nitrogen) or oxygenated for at least 30 min. Furthermore, during performing the electrochemical measurements nitrogen (or oxygen) over the solution was kept. All experiments were carried out at room temperature.

The source of external magnetic field was utilized the neodymium permanent magnet with an intensity of ca. 140 mT. The shape of the magnet was selected to reach the uniform intensity distribution of the magnetic field within the electrochemical cell.

The thermal properties of catalyst precursor were investigated in temperature range from room temperature to 1000 °C by a thermal gravimetric analyser (TA Instruments Q50 Thermal Gravimetric Analyzer, New Castle, DE, USA). The measurements were performed by utilizing samples of 5 mg with heating rate of 5 K min^−1^ under high purity nitrogen.

The crystallographic structure of catalysts was characterized by XRD measurements using a Bruker D8 Discover diffractometer (Bruker, Bremen, Germany) equipped with sample stage, sealed tube with Cu anode (1.5406 Å), Goebel focusing mirror (Bruker, Bremen, Germany, and LynxEye detector (Bruker, Bremen, Germany). The data acquisition was recorded up to 2θ equal 90°.

For TEM sample preparation, a diluted suspension of catalysts was introduced onto the Cu grid contained a Formvar film (Agar Scientific, Stansted, UK). The TEM images were performed on a Talos F200X HRTEM microscope (ThermoFisherScientific, Hillsboro, OR, USA) equipped with four detector super-EDS systems (FEI). Each sample was examined in varied grid places to receive the presentable characterization of the catalytic materials.

The Raman spectrums were measured with the DXR Raman spectrometer (Thermo Scientific, Waltham, MA, USA with 50×/NA 0.75 objective, which utilizes an excitation laser having a wavelength of 532 nm. Calibration procedure was carried out utilizing a silicon wafer at 520 cm^−1^ Raman signal.

Moreover, the samples of catalytic materials were tested using ^57^Fe Mössbauer spectroscopy (home-made Mössbauer spectrometer—designed and built by Łukasiewicz Research Network–Institute of Microelectronics and Photonics, Warsaw, Poland). Experiments were carried out at room temperature with the application of a ^57^Co-in-Rh source.

## 3. Results and Discussion

### 3.1. Physicochemical Characterization

**Figure 1:**
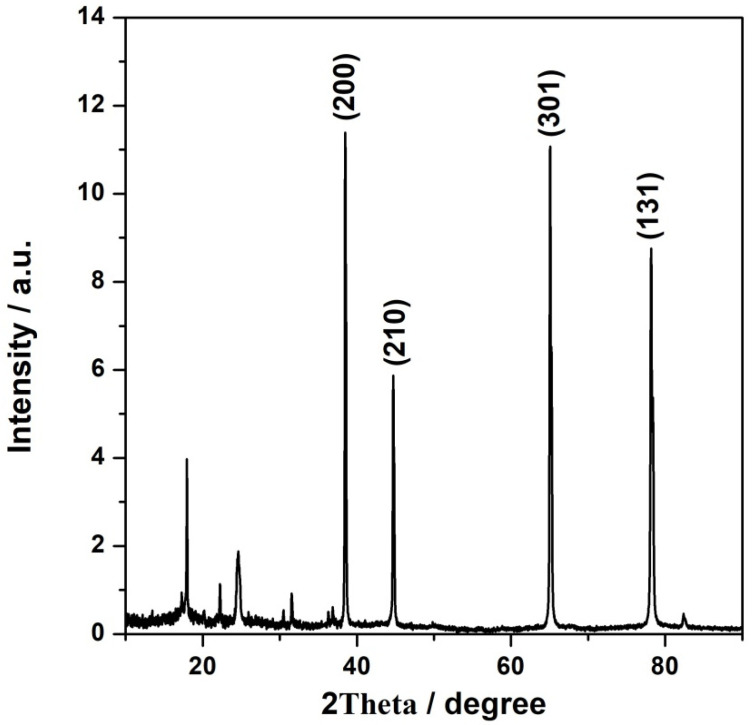
X-ray diffraction patterns for the CFeN@rGO-COOH electrocatalysts.

Powder X-ray diffraction was employed to study the crystal structure of the synthesized catalytic materials. The X-ray diffraction patterns of NFeC@rGO-COOH recorded at a 2θ range from 10° to 90° are displayed in Figure 1. The synthesized catalytic material exhibited a broad diffraction peak at 2θ = 24.7°, which corresponded to the (111) reflection plane, which is characteristic of the rGO_COOH carbon support [45]. After pyrolysis, the diffraction patterns of the catalyst (NFeC@rGO-COOH) appeared at angles of 17.96, 22.22, 30.47, 31.54, 36.84, 38.49, 44.70, 65.07, 78.16, and 82.38. The diffraction peaks at 2θ = 38.49, 44.70, 65.07, and 78.16 were related to the (200), (210), (301), and (131) reflections, respectively, confirming the partial decomposition of PB during calcination, as well as the presence of additional coexisting phases Fe_x_C with the PB [46]. The obtained diffraction peaks are narrow, indicating the system’s crystalline structure and the considerably large sizes of PB.

**Figure 2:**
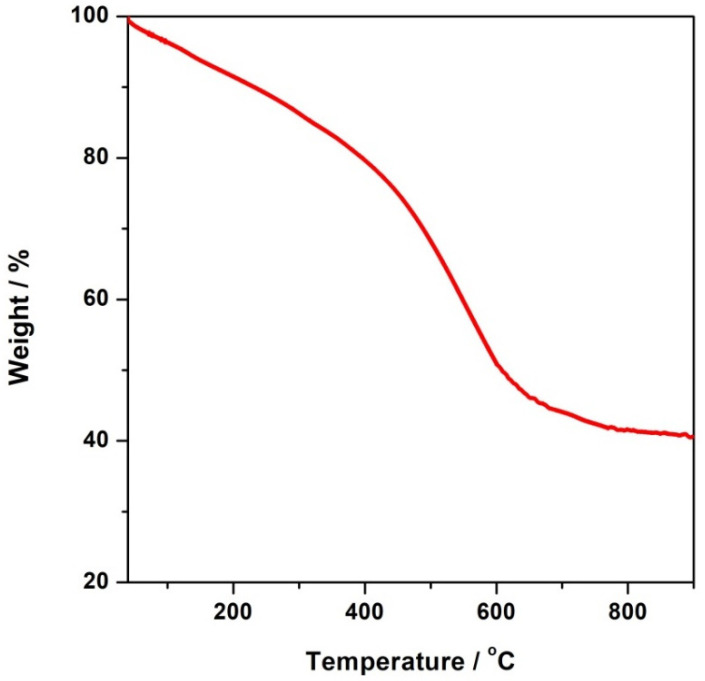
TG profile of CFeN@rGO-COOH.

The TG curve the CFeN@rGO-COOH catalyst sample under N_2_ from 25 to 900 °C is shown in Figure 2. The corresponding measurements displayed a gradual weight loss in the range of 25 to 400 °C, which accounted for ~20% of the initial weight loss, which was due to the loss of adsorbed and coordinated water molecules. The notable mass loss observed above 400 °C was related to the decomposition of CFeN@rGO-COOH, indicating the Prussian blue was thermally stable up to 400 °C, which is more stable than analogous hexacyanometallates [47]. Therefore, the calcination of Prussian blue at a particular temperature has an impact on the generation of new active catalytic centres due to the partial or complete decomposition of its structure.

**Figure 3:**
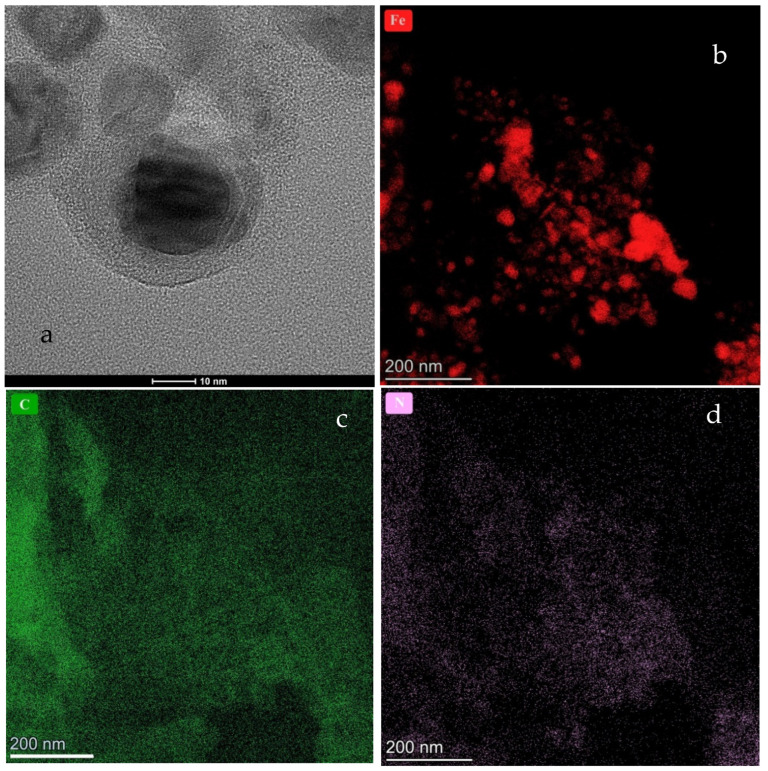
HR-TEM images of the (**a**) CFeN@rGO-COOH sample. Additionally, elementals mapping analysis of (**b**) Fe, (**c**) C, and (**d**) N are displayed.

The HR-TEM images of CFeN@rGO-COOH are presented in Figure 3. These images clearly display the spherical CFeN@rGO-COOH nanostructures as well as rGO sheets which provide an effective conducting structure. Direct observations reveal that the CFeN@rGO-COOH nanoparticles were covered in a carbon-coating to form core–shell nanoparticles. Detailed analysis of HR-TEM images of the dark core exhibited lattice fringes with a spacing of 0.24 nm, corresponding well to the (210) plane of Fe_x_C. Figure 3b–d show the distribution of the crucial elements responsible for the electrochemical activity towards the ORR (Fe, C, and N). The homogenous distribution of nitrogen and carbon were observed, whereas iron showed that the CFeN nanostructures were spherical and non-homogeneously dispersed.

**Figure 4:**
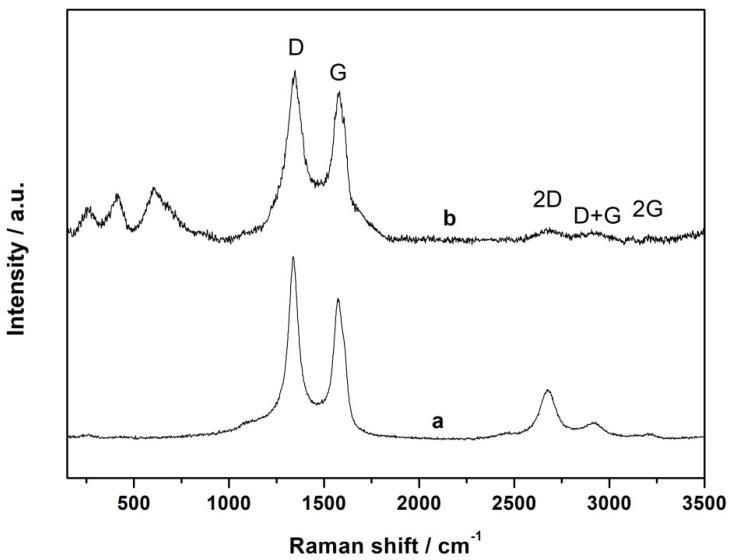
The Raman spectra of rGO-COOH (a) and CFeN@rGO-COOH (b).

Raman spectroscopy was utilized to obtain information about the atomic structure of carbon materials (e.g., graphene layer extension and degree of disorder) in the catalytic systems [48]. In a representative Raman spectrum, shown in Figure 4, the 2 typical peaks around 1340 cm^−1^ and 1574 cm^−1^ are attributed to disordered carbon (D band) and ordered graphite (G band), respectively. These bands are associated with vibrations inside and on the edges of graphene layers. The ratio of the intensity of the D to G band (I_D_/I_G_) is commonly used as an indicator of the degree of surface defects in graphene-based materials. The I_D_/I_G_ value of the CFeN@rGO-COOH sample wa of 1.06, suggesting a high graphitization degree of the synthesized material (for pristine rGO-COOH, I_D_/I_G_ = 1.3). The analysis of the higher-energy region showed that additional well-defined 2D, D + G, and 2D’ bands appeared, confirming less disorder [49]. Furthermore, the Raman spectrum of CFeN@rGO-COOH did not contain characteristic vibrational bands for CN groups (2050–2200 cm^−1^) which were recorded in the spectrum of freshly prepared Prussian blue [50,51]. Additionally, other characteristic signals appeared in the lower part of the spectrum at 607, 406, and 249 cm^−1^ that can be assigned to Fe–C and Fe–C–N vibration bands and C–Fe–C deformation band [52]. This also indicates that during pyrolysis, Prussian blue completely decomposed and provided the C and N sources to form Fe–C–N catalytic centres.

**Figure 5:**
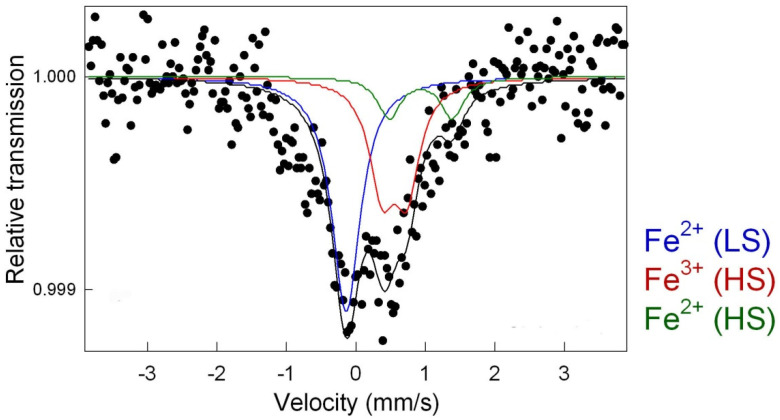
Room-temperature Mössbauer spectra of sample CFeN@rGO-COOH.

Finally, the active sites of thre iron-based catalysts were analysed by utilizing Mössbauer spectroscopy at room temperature. The analysis of the Mössbauer spectrum for thermally treated, Prussian-blue-supported rGO was performed by fitting two doublets D1 and D2 and one singlet (Figure 5). Furthermore, the identification of appropriate iron species was performed based on correlating the obtained Mössbauer spectra to the literature data. The existence of a singlet with a slight isomer shift (IS = −0.03 mm s^−1^) near zero and the lack of a quadrupole splitting (QS) represented the Fe^2+^ cation with a low-spin state [46]. This can be interpreted as super-paramagnetic iron or iron carbides preserved by the carbon matrix. The latter was confirmed in the XRD pattern and TEM images. Furthermore, the doublet D1 with an IS of 0.60 mm s^−1^ and QS of 0.36 mm s^−1^ was assigned to Fe^3+^ (HS), indicating a change in the environment of the high-spin state of iron [53]. The second doublet D2 (IS = 1.1 mm s^−1^, QS = 1.0 mm s^−1^) represented high-spin state Fe^2+^ [54]. Both doublets D1 and D2 were correlated with iron atoms that were octahedrally coordinated with carbon and nitrogen atoms, respectively.

**Figure 6:**
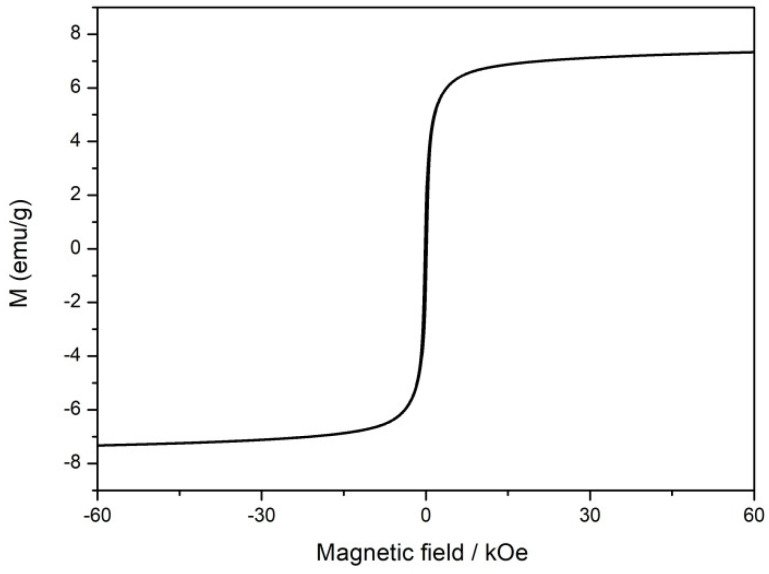
Magnetic hysteresis loops of the CFeN@rGO-COOH sample.

The existence of iron can endow a catalyst with paramagnetic properties. The magnetization hysteresis curves of the annealed catalyst under an appropriate magnetic field range at room temperature were measured to check its magnetic characteristics. The tested catalyst exhibited standard ferromagnetic hysteresis loops due to the presence of metallic iron nanoparticles (Figure 6). The saturation magnetization of CFeN@rGO-COOH was determined to be 7.5 emu g^−1^, which is lower than pristine iron whose saturation magnetization can reach 220 emu g^−1^ [55]. The lower saturation magnetization value of the annealed catalyst may indicate less metallic iron [56]. The magnetic properties of the annealed catalyst (CFeN@rGO-COOH) were connected with its catalytic activity and ability to increase oxygen transportation.

### 3.2. Electroreduction of Oxygen and Diagnosis Measurements

**Figure 7:**
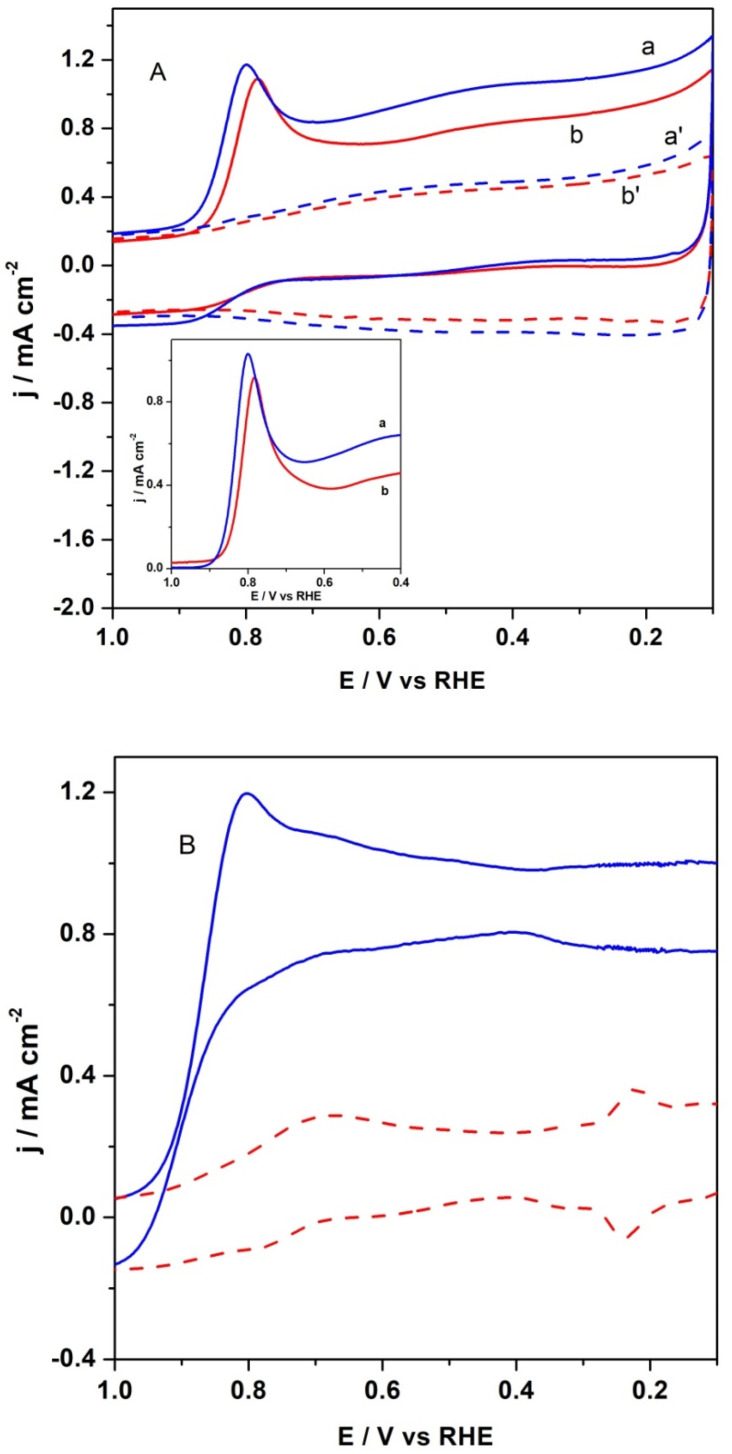
(**A**) Cyclic voltametric characterization of CFeN@rGO-COOH recorded in (b) O_2_-saturated (solid red curve) and (b’) deaerated (dash red curve) in the absence of external magnetic field. Cyclic voltametric responses of CFeN@rGO-COOH recorded in (a) O_2_-saturated (solid blue curve) and (a’) deaerated (dash blue curve) in the presence of external magnetic field. Insert background subtracted cathodic peak (a) with and (b) without external magnetic field. (**B**) Cyclic voltametric characterization of Pt/C nanoparticles recorded in O_2_-saturated (solid blue curve) and deaerated (dash red curve). Electrolyte 0.1 mol dm^−3^ KOH. Scan rate: 50 mV s^−1^.

The cyclic voltametric (CV) curve of CFeN@rGO-COOH catalyst in a deoxygenated electrolyte is shown in Figure 7, which exhibits mainly a rectangular shape with high background current density, is a typical attribute of carbonaceous materials. Furthermore, a small and poorly defined reduction peak appeared in the potential range of 0.7–0.8 V. The catalytic materials in the presence of an external magnetic field showed slightly higher background currents (curves b’ and a’ in Figure 7). For the reference catalyst (20% Pt/C), not show here, the same measurement was performed. The electrochemical behaviour of Pt/C nanoparticles in alkaline media in the presence of external magnetic field was similar to that in its absence.

To further elucidate the ORR activity of the obtained catalyst, the CV experiments in O_2_-saturated 0.1 mol dm^−3^ KOH at room temperature in the absence and presence of an external magnetic field were performed. The presence of a magnetic field increased peak current of the oxygen reduction over the CFeN@rGO-COOH catalyst. The peak potential also shifted positively by 17 mV (Figure 7A). As shown in Figure 7A (inset), the background-corrected voltametric responses with CFeN@rGO-COOH catalyst in O_2_-saturated 0.1 mol dm^−3^ KOH solution in the presence and absence of external magnetic field displayed a well-defined cathodic peak, confirming the catalytic properties towards the ORR. As a comparison, the CV curve of commercial Pt/C reference material is presented in Figure 7B. It illustrates that the ORR peak for CFeN@rGO-COOH appeared at a lower potential (about 50 mV) compared with the benchmark 20 wt% Pt/C catalysts, indicating the similar catalytic activity of CFeN@rGO-COOH in alkaline media.

**Figure 8:**
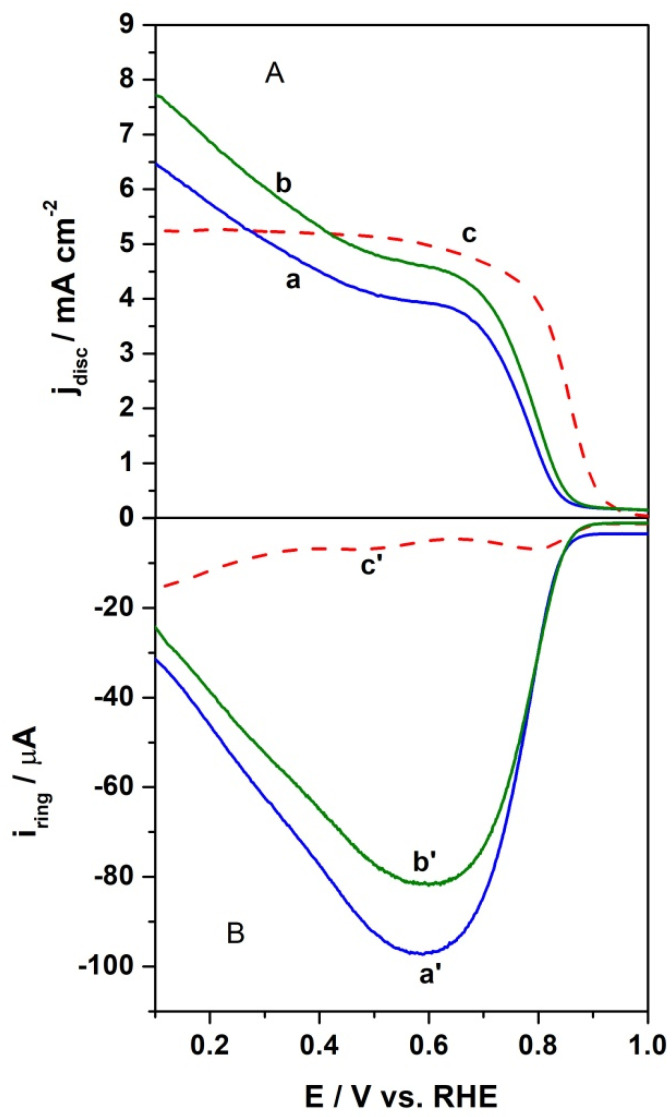
RRDE polarization curves (background subtracted) for oxygen reduction at CFeN@rGO-COOH (a) in the absence of external magnetic field (blue curve) and (b) in the presence of external magnetic field (green curve), and (c) Pt/C nanoparticles (red curve). (**A**) Disk currents and (**B**) ring currents. Electrolyte: O_2_-saturated 0.1 mol dm^−3^ KOH. Scan rate: 10 mV s^−1^. Rotation rate: 1600 rpm. Ring currents are obtained upon application of 1.21 V.

To study the catalytic activity of the proposed catalyst towards the ORR, the linear sweep voltammetry measurements using RRDE were carried out in an oxygen-saturated alkaline electrolyte solution at a 10 mVs^−1^ scan rate and a rotation rate of 1600 rpm with a constant ring current of 1.2 V. The obtained potentiodynamic response for the investigated catalytic material were distinguished by a wave-type curve, suggesting that the electrocatalytic oxygen reduction was mainly controlled by a one-step, four-electron pathway. The polarization curves comparing the activities of the proposed catalyst with the benchmark Pt/C catalyst are shown in Figure 8. It is commonly accepted that the onset potential is indicative of the activities of electrocatalytic materials, e.g., towards the ORR. It can be seen that the onset potential for the layer containing annealed CFeN@rGO-COOH in the absence of an external magnetic field was 0.85 V, whereas the application of magnetic field positively shifted its potential by ca. 15 mV. This is still about 50 mV lower than the onset potential of benchmark Pt/C. This indicates that the CFeN@rGO-COOH catalyst shows a higher electrocatalytic activity towards the ORR under an external magnetic field. It should be noted that the onset potential for the ORR at catalytic layers containing CFeN@rGO-COOH nanoparticles under RRDE experiments was evaluated based on the current densities, at which they approached 5% of the recorded limiting current [57]. Moreover, the half-wave potential (*E*_1/2_) was determined at 0.76 V for CFeN@rGO-COOH in the absence of an external magnetic field, at 0.78 V in the presence of an external magnetic field, and at 0.85 V for the benchmark Pt/C nanoparticles. The slightly lower onset potential values indicate a higher activation energy due to sluggish ORR kinetics. Furthermore, the lower *E*_1/2_ of CFeN@rGO-COOH was related to the thicker layer after modification with respect to the reference catalytic material. The current densities for the studied catalyst in the case of an applied external magnetic field were more pronounced than in its absence. The application of the magnetic field significantly increased the transport of oxygen to the catalyst surface [58]. These results indicate that the reduction currents were controlled by a nearly identical mechanism in the presence and absence of an external magnetic field, i.e., approaching a 4-electron mechanism, which is commonly accepted to be the case for benchmark Pt/C in an alkaline solution.

To obtain more information about the oxygen reduction over the CFeN@rGO-COOH catalyst, RDE polarization curves at different rotation rates (400–2500 rpm) and in the presence and absence of an external magnetic field were collected (not shown here). The recorded RRDE curves were utilized to perform the Levich and Koutecky–Levich (*K*-*L*) analysis to compare the selectivity of the electroreduction of O_2_ to H_2_O using the proposed catalyst both in the presence and absence of an external magnetic field. The dependencies of the RDE current densities (at 0.55 V) were plotted vs. the square root of the rotation rate (Levich plots—not shown here) for the catalytic layer of CFeN@rGO-COOH. Linear responses were observed (i.e., typical for systems limited only by the diffusion of oxygen in an electrolyte) both in the presence and absence of a magnetic field. Using a potential of 0.75 V, the layer of catalytic material in both environments gave a nonlinear response.

**Figure 9:**
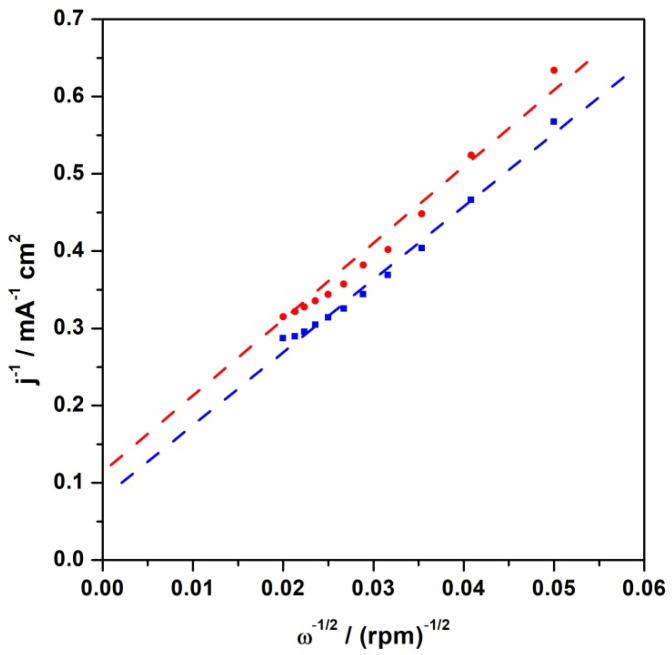
Koutecky–Levich reciprocal plots for electroreduction of oxygen (at 0.75 V) at CFeN@rGO-COOH with (blue curve) and without (red curve) external magnetic field.

The RRDE polarization curves were also analysed by utilizing the Koutecky–Levich equation, assuming that it is only valid in the mixed diffusion and kinetic-limited region, i.e., 0.75 V. For this purpose, utilizing reciprocal coordinates yielded non-zero intercepts, clearly showing the kinetic limitations of the electrocatalytic layer. The corresponding Koutecky–Levich plots are shown in Figure 9. The Koutecky–Levich equation can be used to obtain kinetic current density values from the experimental data, as follows:

$$\frac{1}{j}=\frac{1}{j_{k}}+\frac{1}{j_{L}}=\frac{1}{nFk_{het}C_{O_{2}}^{o}}+\frac{1}{j_{L}}$$

where: *j*—experimental current density; *j_k_*—kinetic current density; *j_L_*—diffusion limiting current density (described by the Levich equation); *k_het_*—the electron transfer heterogeneous rate constant (cm s^–1^); $C_{O_{2}}^{o}$—the bulk reactant concentration (oxygen in electrolyte: 1.2 × 10^−3^ mol dm^−3^) [59]. The Koutecky–Levich plots (Figure 9) are linear, implying that the proposed catalytic system is well-behaved, and the reaction is in agreement with the well-known (mainly four-electron) mechanism. The kinetic current density determined from the intercept of the linear fit of the *K*-*L* plot at 0.75 V (vs. RHE) with the x-axis was utilized to estimate the kinetic parameter (*k_het_*). The results indicated the internal rate of heterogeneous charge transfer in the absence and presence of an external magnetic field. The evaluated rate (in heterogeneous units) for oxygen reduction over CFeN@rGO-COOH without a magnetic field was 7.1 × 10^−2^ cm·s^−1^ and 8.9 × 10^−2^ cm·s^−1^ with a magnetic field, which was compared with the value obtained for the reference catalyst (Pt nanoparticles)—1.1 × 10^−1^ cm·s^−1^.

**Figure 10:**
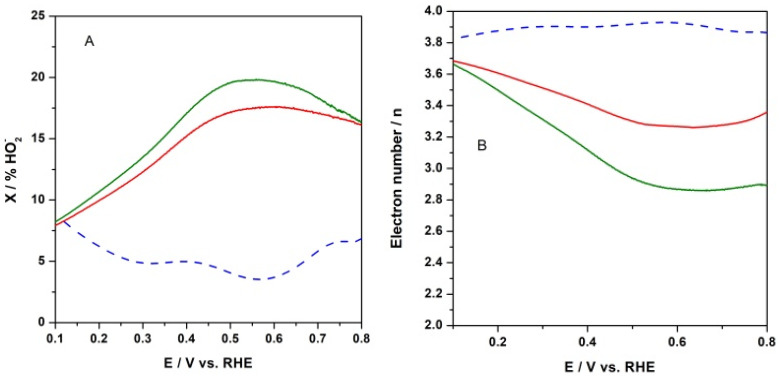
(**A**) Percent fraction of peroxide anion (X% HO_2_^−^) formed during electroreduction of oxygen at CFeN@rGO-COOH in (a) the absence (solid green curve) and (b) the presence (solid red curve) of an external magnetic field, and Pt/C nanoparticles (dash blue curve) under the conditions of RRDE voltametric experiment. (**B**) Numbers of transferred electrons (n) per oxygen molecule during electroreduction of oxygen under conditions described above.

Since the peroxide anion has been identified as an intermediate species during the ORR, the formation of peroxide species (expressed as % HO_2_^−^) was monitored from the recorded RRDE profiles. The relative concentration of formed peroxide anion with respect to the total ORR products was calculated according to the following equation:

$$X_{\%OH_{2}^{-}}=\frac{2\cdot \frac{I_{ring}}{N}}{I_{disk}+\frac{I_{ring}}{N}}\;\cdot 100$$

where *I_ring_* and *I_disk_* stand for ring and disk current, respectively, and N is the collection efficiency [60]. As can be seen in Figure 10A at the electrode with CFeN@rGO-COOH catalyst, the HO_2_^−^ production was higher than that of the reference catalyst (Pt/Vulcan) in the examined potential range. The peroxide species formation increased up to ca. 20% in the absence of an external magnetic field upon applying a potential ranging from 0.8 V to 0.65 V. The introduction of an external magnet decreased the side products, peroxide anions, by up to 16% for CFeN@rGO-COOH in the same potential range, whereas at potentials lower than 0.5 V (the diffusion-controlled current region), the calculated fractions of peroxide species decreased by ca. 8% at E = 0.1 V for the studied catalyst, while it increased to ca. 8% for the reference Pt/C at the same potential (E = 0.1 V). These results reveal that the ORR involved mainly the four-electron pathway.

Furthermore, based on the RRDE measurements (Figure 10B), the number of electrons exchanged per oxygen molecule (*n*—defined as the arithmetic average) were thereafter computed as a function of the applied potential, utilizing the following equation:

$$n=\frac{4I_{disk}}{I_{disk}+\frac{I_{ring}}{N}}$$

The oxygen reduction process at CFeN@rGO-COOH in the absence of an external magnetic field yielded between 3 and 3.6 electrons, which was less than the value obtained for the same catalyst upon applying an external magnetic field (from 3.4 to 3.7). In all cases, the electron transfer numbers were lower than those observed for benchmark Pt/C (3.8–3.9). The calculated electron transfer numbers for both catalytic materials were generally in good agreement with the percentage of peroxide species. Moreover, these results indicated that the CFeN@rGO-COOH catalyst could be classified as a 4-electron oxygen reduction catalyst.

## 4. Conclusions

Here, we prepared a non-precious iron electrocatalyst by calcining Prussian blue immobilized onto carboxylic-acid-functionalized reduced graphene oxide. The electrochemical diagnostic experiments clearly demonstrated that heat treatment (at moderate temperatures) of Prussian blue, dispersed over the carboxylic-acid-functionalized rGO (support), displayed catalytic active sites towards the oxygen reduction reaction in alkaline media. We also showed that the efficiency of the iron-based catalyst towards the ORR was improved by introducing an external magnetic field. The analysis of the surface chemical state showed that the presence of Fe_x_C may be a key point to enhance the active species, improving the ORR by accelerating charge transfer. Furthermore, the formation of peroxide species was slightly higher than that at the Pt/C reference catalyst (>0.5 V), as determined by RRDE. These exceptional properties demonstrate the promise of our proposed strategy for producing enhanced electrocatalysts for oxygen reduction based on calcining metal hexacyanometallates, deposited on reduced graphene oxide. Further research will be performed along this line.

## Data Availability

Not applicable.
